# Neuroprotective effects of pomegranate (*Punica granatum* L.) juice and seed extract in paraquat-induced mouse model of Parkinson’s disease

**DOI:** 10.1186/s12906-021-03298-y

**Published:** 2021-04-26

**Authors:** Samah M. Fathy, Heba A. El-Dash, Noha I. Said

**Affiliations:** Zoology Department, Faculty of Science, Fayoum University, Fayoum, Egypt

**Keywords:** Apoptosis, Inflammation, Oxidative stress, Paraquat, Parkinson’s disease, Pomegranate

## Abstract

**Background:**

Paraquat, (PQ), an herbicide that can induce Parkinsonian-like symptoms in rodents and humans. The consumption of phytochemical-rich plants can reduce the risk of chronic illnesses such as inflammation and neurodegenerative diseases. The present study aimed to investigate the protective effects of pomegranate seed extract (PSE) and juice (PJ) against PQ-induced neurotoxicity in mice.

**Methods:**

Mice were assigned into 4 groups; three groups received PQ (10 mg/kg, i.p.) twice a week for 3 weeks. Two of the PQ-induced groups pretreated with either PSE or PJ. Detection of phytochemicals, total phenolics, and total flavonoids in PSE and PJ was performed. Tyrosine hydroxylase (TH) level was measured in the substantia nigra (SN) by Western blotting technique. Striatal dopamine (DA) and 3,4-dihydroxyphenylacetic acid (DOPAC) were detected using high-performance liquid chromatography (HPLC). The levels of adenosine triphosphate (ATP), malondialdehyde (MDA), and the activity of the antioxidant enzymes were estimated in the striatum by colorimetric analysis. Striatal pro-inflammatory and anti-inflammatory markers using enzyme-linked immunosorbent assay (ELISA) as well as DNA fragmentation degree by qualitative DNA fragmentation assay, were evaluated. Real-time polymerase chain reaction (qPCR) assay was performed for the detection of nuclear factor kappa B (NF-кB) gene expression. Moreover, Western blotting analysis was used for the estimation of the cluster of differentiation 11b (CD11b), transforming growth factor β (TGF-β), and glial cell-derived neurotrophic factor (GDNF) levels in the striatum.

**Results:**

Pretreatment with PSE or PJ increased the levels of TH in the SN as well as DA and its metabolite in the striatum that were reduced by PQ injection. PSE and PJ preadministration improved the PQ-induced oxidative stress via a significant reduction of the MDA level and the augmentation of antioxidant enzyme activities. PSE and PJ also significantly downregulated the striatal NF-кB gene expression, reduced the PQ-enhanced apoptosis, decreased the levels of; pro-inflammatory cytokines, CD11b, and TGF-β coupled with a significant increase of; interleukin-10 (IL-10), GDNF, and ATP levels as compared with PQ-treated mice.

**Conclusions:**

The current study indicated that PSE and PJ consumption may exhibit protective effects against PQ-induced neurotoxicity in mice.

**Supplementary Information:**

The online version contains supplementary material available at 10.1186/s12906-021-03298-y.

## Background

Parkinson’s disease (PD) is a chronic neurodegenerative disease [1], affecting mainly old people with enormous impacts on their life [2]. PD is a multisystem disorder mainly characterized by depletion of the dopaminergic neurons in the substantia nigra pars compacta (SNpc) with subsequent dopamine (DA) deficiency in the dorsal striatum [3]. Dopaminiergic neuronal loss was observed to be associated with declined protein and activity levels of tyrosine hydroxylase (TH) [4]. It is well known that TH is the rate-limiting enzyme for DA synthesis which plays a crucial role in the disease progression [5]. It has been reported that DA is controlled by TH and it interacts with a-synuclein protein leading to the formation of intra-neuronal inclusions, named Lewy bodies (LBs) with consequent apoptosis [5]. LBs formation was associated with a movement impairment and a group of motor and non-motor symptoms [6]. The molecular pathogenesis of PD also involves calcium release dysfunction, mitochondrial dysfunction, oxidative stress, and neuroinflammation [7].

Recent studies reported the participation of the environment to PD pathogenesis [8] while a small portion of PD incidence is correlated with genetic factors such as α-synuclein and parkin gene mutations. Moreover, an epidemiological survey suggested that the exposure to herbicides may induce PD risk [9].

Paraquat (PQ, 1, 1 ‘dimethyl-4, 4’-bipyridinium) is the most common herbicide that is used in the agricultural sector worldwide for weed control [10, 11]. PQ occurs naturally as a divalent cation PQ^2+^. When PQ^2+^ is injected into mice for at least 3–4 weeks, it induces a loss of dopaminergic neurons [12]. In the brain, PQ^2+^ undergoes redox cycling in the presence of NADPH hydroxylase on the microglia and is reduced to the monovalent cation PQ^+^. PQ^+^ is a DA transporter substrate and is accumulated in the dopaminergic neurons where it starts a new redox cycle intracellularly. Subsequently, superoxide anions are generated and reactive oxygen species (ROS) are produced in the dopaminergic neurons leading to oxidative stress-related cytotoxicity and neurotoxicity [13]. Consequently, PQ accumulation has been ascribed with damaged dopaminergic neurons through oxidative stress which increases the neuroinflammatory process in rat and mouse brains [14, 15].

ATP exerts a vital role in the exchange of energy in various biological systems. Moreover, it is found in every metabolically active cell. Therefore, the measurement of ATP levels can be used as an indicator of the functional integrity of dopaminergic neuronal cells in the dorsal striatum [16].

It has been reported that glial cell-derived neurotrophic factor (GDNF) exhibits a prominent neurorestoration and neuroprotection in multiple neurodegenerative diseases, including PD. [17] It was recorded that GDNF is a crucial factor for the survival of the dopaminergic neurons [18]. It was investigated in different PD clinical trials since the main hallmark for PD pathology is the dopaminergic neuronal loss in the brain [18]. Subsequently, it was vital to be assessed in the current animal model of PD and might be used as an indicator for the degeneration of dopamine neurons.

Despite the pharmaceutical treatments of PD may reduce the symptoms, they exhibit severe adverse effects that preclude their long-term use [19] and it is necessary to ameliorate the complications accompanying PD with the least possible side effects. Subsequently, non-pharmaceutical interventions are emerging to overcome drug side effects in PD individuals.

Naturally occurring phytochemicals have been extensively used nowadays for the protection of the body from oxidative damage ascribed with free radicals, neuroinflammation, and DNA fragmentation especially in the brain tissue [20]. It has been found that pomegranate (*Punica granatum* L.) fruit, with various groups of phytochemicals, exhibits many therapeutic properties including antioxidant, anti-inflammatory, anti-proliferative, anti-cancer, antimicrobial, neuroprotective, and anti-apoptotic consequences [21, 22]. Noteworthy, the utilization of pomegranate was recommended by researchers due to the abovementioned medicinal properties [23]. Phenolic acids and flavonoids’ components of polyphenols detected in pomegranate were associated with its neuroprotective impact in mice model of Alzheimer’s disease [24]. Despite all the available information on the neuroprotective influences of phytochemicals on neurodegenerative diseases, the effect of pomegranate against PD is based on very limited data [25].

Up to our knowledge, the current study is the first-ever to assess the potential antioxidant, anti-inflammatory, and anti-apoptotic capabilities of pomegranate juice (PJ) and seed extract (PSE) against PQ-induced Parkinsonian mice model.

## Methods

### Chemicals

PQ dichloride (≥ 98%) was bought from Sigma (St. Louis, MO, USA). The rest of the chemicals were of the highest analytical grade.

### Plant material

Pomegranate (*Punica granatum* L.) fruits were reaped at Fayoum City, Egypt in the months of September–October, 2019. The coordinates of the city are latitude 16° 53′ 12.59“ N and longitude: 42° 33’ 23.99” E following the degree minutes second (DMS) system. The plant material was authenticated by comparing it with the well-known herbarium specimens found at the Herbarium of Flora Researches Centre, Agriculture museum campus (CAIM), Dokki, Giza, Egypt.

### Preparations of PSE and PJ

Fresh red pomegranate fruits were washed and manually peeled. The seeds were squeezed and pulsed in juicer-blender. The juice was filtered from the cell debris and kept at − 20 °C. The dried materials of seeds remained after juice preparation was allowed to dry and then ground forming a powder. Thereafter, 500 g of this powder was extracted in absolute ethanol (1:10 w/v) at 25 °C for 24 h. The mixture was then filtered by 0.45 μ pore size filters. The ethanol was completely evaporated using a rotary vacuum evaporator at 40 °C and the seed extract was kept at − 20 °C until use.

### Gas chromatography–mass spectrometry (GC-MS) analysis

The GC-MS technique was carried out using a GC (Agilent Technologies 7890A) edged with a mass-selective detector (MSD, Agilent 7000 Triple Quad) supplemented with an apolar Agilent HP-5 ms (5%-phenyl methyl poly siloxane) capillary column (30 m × 0.25 mm i. d. and 0.25 μm film thickness). Helium was the carrier gas with a linear velocity of 1 ml/min. The temperatures of the injector and the detector were adjusted at 200 °C and 250 °C, correspondingly. Split injection mode and the MS operating factors were adjusted according to Fathy and Drees [26]. The components were detected by comparing their mass spectra and retention time with those of the reliable compounds and by computer equalizing with NIST and WILEY library as well as by contrasting the fragmentation pattern of the mass spectral information with those documented in the text [27].

### Determination of the total phenolic content (TPC)

TPC was measured in PSE and PJ by using the Folin-Ciocalteu method as described by Velioglu et al. [28]. Gallic acid was the standard solution. Results were expressed as mean value ± standard deviation (S.D.) of three times recurring determinations.

### Determination of total flavonoid content (TFC)

TFC of PSE and PJ was detected by using aluminum chloride (ALCL_3_) colorimetric assay according to Zhishen et al. [29]. The mixture absorbance was captured by the spectrophotometer against blank at 510 nm using catechin as a standard solution. Results were expressed as mean value ± S. D of three times recurring determinations.

### Animals

The current experimental study was performed by following National Health Institute (NIH) guidelines for Ethical Conduct in the Care and Use of Laboratory Animals and approved by the National Organization of Drug Control and Research (NODCAR), Egypt.

Adult male albino mice weighing 20–25 g (8 weeks old) were brought from the National Cancer Institute (NCI), Egypt. Animals were kept in conventional cages (polycarbonate) under standard conditions of ventilation, temperature (25 + 2 °C) and subjected to 12 h light/dark cycle throughout the entire study. They were given water ad libitum and fed with the standard laboratory diet. Mice were allowed to accommodate the laboratory condition 1 week before the beginning of the experiment.

### Experimental groups and animal dosing

The treatment design for the experiment was chosen based on the previous studies that proposed the protective effects of pomegranate against other oxidative stress and inflammatory-related diseases [22, 30]. Mice were randomly divided into four groups of 10 animals each; group 1(control group): animals were treated with 0.9% saline, group 2 (PQ group): animals received PQ in 0.9% saline solution (10 mg/kg, i.p.) two times a week for 3 weeks [31, 32], group 3 (PQ + PSE): animals received PSE (500 mg/kg/day, by gavage) according to Doostan et al. [22] for 2 weeks before PQ injection and then continued daily along with PQ treatment (10 mg/kg, i.p. two times a week) for another 3 weeks, and group 4 (PQ + PJ): each mouse received 5 ml of 1:40 dilution of PJ according to Hartman et al. [24]. PJ was given daily by gavage for 2 weeks prior to PQ treatment and then administrated daily with PQ treatment (10 mg/kg, i.p., twice a week) for further 3 weeks.

### Dissection and sampling

At the end of the study, all mice were euthanized by decapitation under pentobarbital (30 mg/kg, i.p.) anesthesia [33]. The brains were quickly dissected and the tissues of the SN and the dorsal striatum were separated by following the atlas [34]. The dissected SN and striatal tissues were rinsed in ice-cold phosphate-buffered saline (PBS, 0.1 M, pH 7.4) and weighed. For the inflammatory and the biochemical parameters, the tissues were homogenized in PBS and sonicated. The homogenates were centrifuged for 5 min at 5000’g at 4 °C to obtain the supernatant. Protein concentration was measured in the SN and the striatal supernatant by the Bio-Rad protein assay kits (Hercules, CA, USA) according to the Bradford method [35]. The SN supernatant was used for measuring TH whereas the striatal supernatant was used for detecting adenosine triphosphate **(**ATP), malondialdehyde (MDA), antioxidant enzyme activities, inflammatory cytokines, the cluster of differentiation 11b (CD11b), GDNF, and transforming growth factor-β (TGF-β). Part of the separated striatum was stored at − 80 °C until required for DA and 3,4-dihydroxyphenylacetic acid **(**DOPAC) estimation and conducting the laddered DNA fragmentation assay. Another part was kept in RNA lysis buffer for conducting a quantitative polymerase chain reaction assay.

### Determination of DA and DOPAC levels in the striatum

Both DA and DOPAC levels were determined by high-performance liquid chromatography (HPLC) according to Chi et al. [36] with some modifications. Striatum samples were collected on dry-ice, the tissue was weighed out, and it was then homogenized for 1 min in 75% methanol. The whole procedure was carried out on the ice. The homogenate was centrifuged at 12000’g for 4 min at 4 °C. 20 μl of tissue supernatant was filtered then injected into HPLC Agilent 1260 apparatus, RP-C18 column (150 × 4.6mmx5μm), and at wavelength 240 nm. The mobile phase used consists of 0.1 M KH2PO4–acetonitrile (91:9, v/v) and octane sulphonic acid (100 mg/L) adjusted to pH 4.75 (with 0.5 M K2HPO4). The mobile phase then filtered and degassed before use. The flow-rate was 1.0 ml/min. The analytical column was kept at 30 °C.

### Estimation of biochemical parameters

#### Determination of ATP

ATP content was estimated in the striatum as stated by the manufacturer’s protocol using ATP Colorimetric/Fluorometric Assay Kit (S. Milpitas Blvd., Milpitas, CA, USA).

#### Measurement of MDA

MDA is an end product of lipid peroxidation (LPO). It was calculated by using (MDA Colorimetric/Fluorometric Assay Kit; BioVision Incorporated., CA, USA) in the tissue homogenate by the formation of thiobarbituric acid reacting substances (TBARS) following the method of Esterbauer and Cheesem [37].

#### Catalase (CAT) assay

CAT activity was detected using a CAT assay kit (CAT Activity Colorimetric/Fluorometric Assay Kit; BioVision Incorporated., CA, USA) according to the instructions from the producer.

#### Superoxide dismutase (SOD) assay

SOD activity was measured using the SOD Activity Assay Kit (BioVision Incorporated., CA, USA) as stated by the protocol’s instructions.

#### Glutathione peroxidase (GPx) assay

GPx activity was tested using the GPx Activity Colorimetric Assay Kit (BioVision Incorporated., CA, USA) according to the protocol’s instructions. In this assay, GPx diminishes Cumene Hydroperoxide while oxidizing GSH to GSSG. Then it was reduced to GSH with NADPH depletion [38].

### Qualitative DNA fragmentation assay

Apoptotic DNA fragmentation was detected in the striatum according to the protocol described by Sambrook et al. [39]. Briefly, fragments of the striatum were suspended in 100 μl lysis buffer containing 10 mM Tris-HCl, pH 7.4, 10 mM NaCl, 10 mM EDTA, and 1% SDS. The tissue was kept with RNAse (1 h at 37 °C), then with 50 μg/ml proteinase K (at 50 °C overnight), centrifuged at 13,000 rpm for half an hour, and precipitated with absolute ethyl alcohol at − 20 °C. The DNA pellet was then rinsed with 70% ethyl alcohol and dissolved in sterile ddH2O. DNA fragments were detected by running the extracted DNA sample on a 1.5% agarose gel with ethidium bromide at 70 Volt and examined under UV trans-illuminator and finally captured.

### Detection of the inflammatory cytokine levels in the striatum

The levels of tumor necrosis factor (TNF)-α, interleukin (IL)-1β, IL-6, IL-10, were estimated in the tissue supernatant by sandwich enzyme-linked immunosorbent assay (ELISA) kits specific for mice (Merck Millipore, San Francisco, California, USA) following the product’s instruction. The absorbance was detected at 450 nm using the microplate reader.

### Determination of nuclear factor kappa B (NF-кB) gene expression by real-time polymerase chain reaction (qPCR) assay

RNA was isolated from the striatal tissue and qPCR was performed as stated by the instructions from the manufacturer using SV Total RNA Isolation System (Promega Corporation, Madison, WI, USA) and Applied Biosystems StepOne™ (Applied Biosystems, Foster City, CA, USA), correspondingly according to Fathy and Said [40]. The NanoDrop™ 2000/2000c Spectrophotometer (Thermo Scientific, Lo, UK) was selected for detecting the concentration and the quality of the isolated RNA. NF-кB gene was used with a forward primer sequence: 5′-CCCTACGGAACTGGGCAAAT-3′ and a reverse primer sequence: 5′-GCGGAATCGAAATCCCCTCT-3′ [41]. NF-кB relative expression level was normalized based on the housekeeping gene “glyceraldehyde 3-phosphate dehydrogenase (GAPDH)” (the sense sequence: 5′-ATGTGTCCGTCGTGGATCTGAC-3′ and the antisense sequence: 5′-AGACAACCTGGTCCTCAGTGTAG-3′) [42].

### Western blotting analysis

Western blotting technique was performed to measure the protein level of TH in the SN. It was also conducted to estimate the protein expression levels of CD11b, TGF-β, and GDNF in the striatum. Equal amounts of total proteins (30 μg) from the supernatant samples were fractionated by sodium dodecyl sulfate-polyacrylamide gel electrophoresis (SDS-PAGE) and transferred onto polyvinylidene difluoride (PVDF) membranes (Millipore Corp., Bedford, MA, USA). Membranes were blocked, incubated with 1:1000 of the specific primary antibody at 4 °C overnight. Antibodies for TH (Thermo Fisher Scientific, PA5–17800), TGF-β (ID11, Thermo Fisher Scientific, MA5–23795), GDNF (Thermo Fisher Scientific, PA5–89957), and CD11b (Thermo Fisher Scientific, PA5–79532) were used. Afterward, membranes were incubated with 1:5000 of goat anti-rabbit IgG antibody, HRP (Thermo Fisher Scientific, 31,460) or goat anti-mouse IgG, HRP (Thermo Fisher Scientific, 31,430) for 1 h at room temperature. Detection reagents for enhanced chemiluminescence (Millipore, CA, USA) were applied and the bands were detected using a chemiluminescence system (New Life Science Products, Boston, MA, USA).

### Statistical methods

Statistical analysis of data was performed using GraphPad PRISM (version 6.01; Graph Pad Software, USA). Data were expressed as the mean ± standard deviation (SD) and analyzed using one-way analysis of variance (ANOVA) followed by Tukey’s test. Value of *P* < 0.05 among groups was considered statistically significant.

## Results

### GC-MS analysis of PSE

Table 1 shows different phytochemicals that were detected by GC-MS analysis of PSE. Various polyphenols were recorded such as propyl gallate, nobiletin, ellagic acid which is a polyphenolic–like hydrolyzable tannins, vitexin, and isovitexin that belong to the group of flavonoids. Oleic acid is an unsaturated fatty acid. Squalene is an isoprenoid substance and geranyl isovalerate is one of the triterpenoids. The rest of the compounds are flavones which constitute a major class of flavonoids.

**Table 1:** GC-MS analysis of PSE showing different phytochemicals

No.	Compounds	Retention time (min.)	Sum area %	Molar mass (gmol^**− 1**^)
1	2′,5′-dimethoxyflavone	5.043	0.58	282.29
2	7,2′,3′-Trimethoxyflavone	6.528	0.67	312.3
3	3,2′,4′,5′,6-pentamethoxyflavone	8.196	0.46	372.37
4	3,6,2′,3′-Tetramethoxyflavone	8.5	0.22	342.34
5	Nobiletin	11.514	1.12	402.399
6	2′,3′-dimethoxyflavone	11.961	8.11	282.29
7	6,7,3′,4′-Tetramethoxyflavone	12.671	2.29	342.35
8	3,5,7, 3′,4′-pentamethoxyflavone	12.884	1.09	372.37
9	3,2′,4′,5′-Tetramethoxyflavone	13.425	0.49	342.3
10	5-Hydroxy-3′,4′,5′,6,7,8-hexamethoxyflavone	13.79	15.79	418.39
11	Oleic acid	14.824	13.41	282.468
12	Vitexin (Apigenin-8-C-glucoside)	15.016	0.77	432.38
13	Propyl gallate	15.82	0.42	212.20
14	Ellagic acid	17.3	0.6	302.197
15	7,3′,4′,5′-Tetramethoxyflavone	17.883	0.35	342.34
16	3,7,8,2′-Tetramethoxyflavone	18.203	0.24	342.35
17	3,6,3′,4′-Tetramethoxyflavone	18.437	0.5	342.3
18	Isovitexin (Apigenin-6-C-glucoside)	19.573	0.77	432.38
19	Geranyl isovalerate	20.504	1.83	238.37
20	Squalene	21.422	1.09	410.730
21	3,5,7-Trimethoxyflavone	22.062	0.91	312.3
22	3-Hydroxy-7,8,2′-trimethoxyflavone	22.804	2.25	328.31
23	7,8,3′,4′-Tetramethoxyflavone	22.956	0.84	342.3

### GC-MS analysis of PJ

Table 2 shows various phytochemicals that were detected by GC-MS analysis of PJ. Polyphenolic compounds were recorded such as ellagic acid which is a polyphenolic–like hydrolyzable tannins as well as vitexin and isovitexin that belong to the group of flavonoids. Geranyl isovalerate is one of the triterpenoids. Oleic acid and Linoleic acid are unsaturated fatty acids. 1-Hexacosanol and 1-Heptacosanol are primary fatty alcohol. The rest of the compounds are flavones which constitute a major class of flavonoids.

**Table 2:** GC-MS analysis of PJ showing different phytochemicals

No.	Compounds	Retention time (min.)	Sum area %	Molar mass (gmol^**−1**^)
1	2′,5′-dimethoxyflavone	4.084	2.03	282.29
2	3′,4′,5,5′,7-Pentahydroxyflavone	11.358	0.43	302.23
3	Nepetin	11.457	0.5	316.26
4	Geranyl isovalerate	11.608	0.44	238.37
5	Oleic acid	11.838	0.69	282.468
6	7,8-Dihydroxyflavone	12.957	0.6	254.24
7	Vitexin (Apigenin-8-C-glucoside)	13.741	0.6	432.38
8	6-hydroxy-2′-methoxyflavone	13.95	0.52	268.26
9	1-Hexacosanol	14.098	0.77	382.717
10	1-Heptacosanol	14.549	1.52	396.7
11	Linoleic acid	14.737	1.46	280.452
12	3,5,7, 3′,4′-pentamethoxyflavone	14.893	0.87	372.37
13	Isovitexin (Apigenin-6-C-glucoside)	15.521	0.5	432.38
14	5-hydroxy- 3′,4′,-5′,6,7,8 hexamethoxyflavone	15.709	0.61	418.39
15	Ellagic acid	17.3	0.89	302.197
16	3,2′,4′,5′-Tetramethoxyflavone	17.711	0.8	342.3
17	7,8,3′,4′-Tetramethoxyflavone	23.025	0.45	342.3

### TPC and TFC in PSE and PJ

It was found that TPC in PSE is 655.00 ± 11.36 mg of gallic acid equivalents/100 g dry weight (DW) whereas TPC in PJ is 241.1 ± 9.99 mg of gallic acid equivalents/100 ml of juice.

Regarding TFC, it was found to be 145.70 ± 6.65 mg of catechin equivalents/100 g DW of PSE whereas TFC in PJ is 39.40 ± 4.26 mg of catechin equivalents/100 ml of the juice.

### Effect of PSE and PJ pretreatment on TH level in the SN of PQ-treated mice

**Fig. 1:**
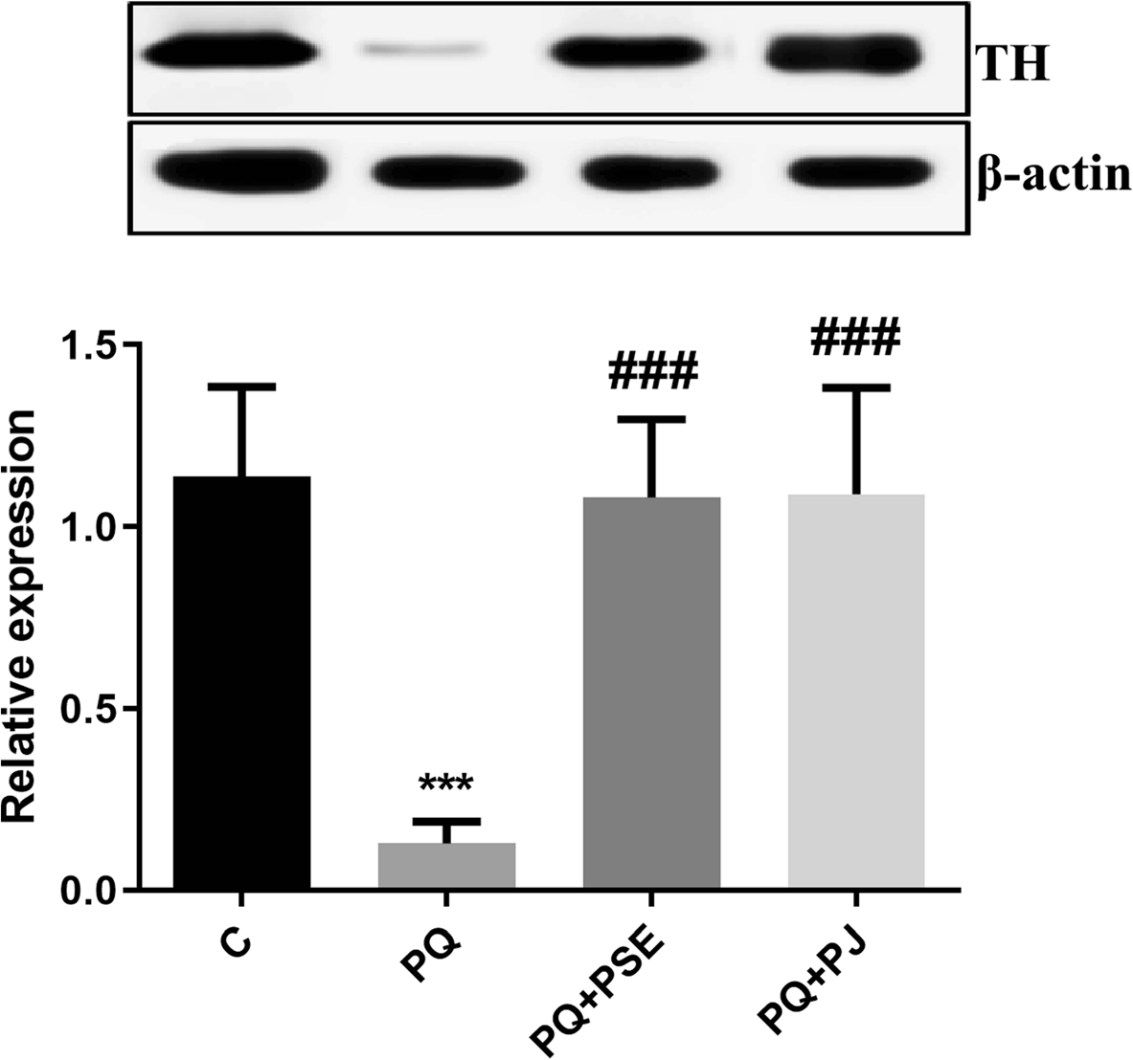
Representative cropped blot with relative expression level of TH in the SN of different animal groups. The full length blots are presented in supplementary figure 1. C = control group; PQ = Paraquat (alone)-induced group; PQ+PSE = PQ-induced group treated with PSE; PQ+PJ = PQ-induced group treated with PJ. Data are expressed as mean ± S.D. of 10 mice in each group. ***: *P* < 0.001 compared with control group; ###: *P* < 0.001 compared with PQ group

The protein level of TH was measured in the SN to check the dopaminergic neuronal loss at different experimental groups. TH level was significantly diminished in the SN region of the PQ (alone)-induced group when compared with the control group (*P* < 0.001). Oral treatment of either PSE or PJ led a remarkable increase in the protein expression of TH (*P* < 0.001) when compared with the PQ (alone)-treated mice (Fig. 1).

### Impact of PSE and PJ administration on the striatal DA and DOPAC levels in PQ-treated mice

**Fig. 2:**
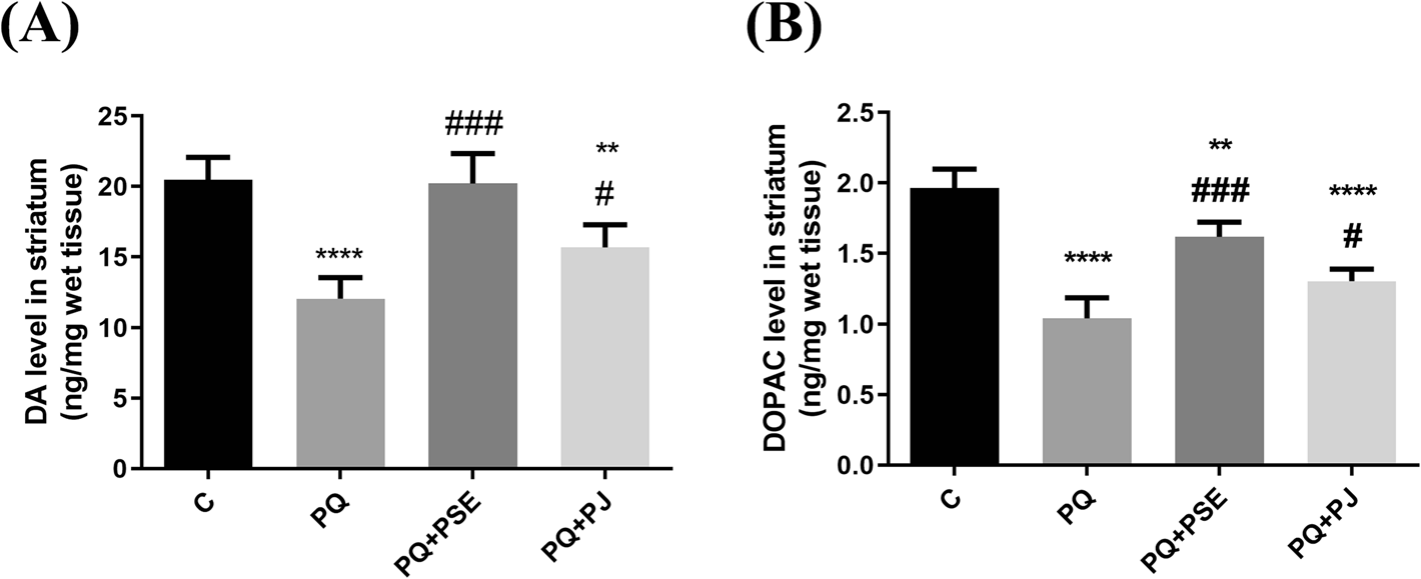
Effect of PSE and PJ supplementation on the striatal levels of DA and its metabolite; DOPAC in PQ-injected animals. Levels of DA (**a**) and DOPAC (**b**) in striatum. C = control group; PQ = Paraquat (alone)-induced group; PQ+PSE = PQ-induced group treated with PSE; PQ+PJ = PQ-induced group treated with PJ. Data are expressed as mean ± S.D. of 10 mice in each group. **: *P* < 0.01 and ****: *P* < 0.0001 compared with control group; #: *P* < 0.05 and ###: *P* < 0.001 compared with PQ group

PQ treatment led to a highly significant reduction in the striatal DA and DOPAC levels when compared with the control mice (*P* < 0.0001). Pretreatment with PSE was associated with a significant elevation in DA and DOPAC levels (*P* < 0.001) when compared with PQ (alone)-induced mice. Administration of PJ also led to a conceivable increase in the levels of DA and DOPAC as compared to PQ (alone)-treated mice (*P* < 0.05; Fig. 2).

### Effect of PSE and PJ preadministration on ATP level in PQ-induced animals

**Fig. 3:**
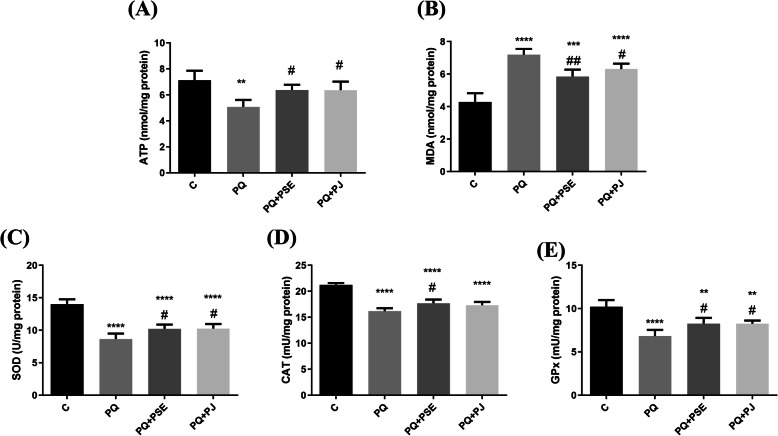
Effect of PSE and PJ supplementation on the striatal levels of ATP, MDA, and antioxidant enzymes’ activity in PQ-induced animals. ATP level (**a**), MDA level (**b**), SOD activity (**c**), CAT activity (**d**), and GPx activity (**e**) in striatum. C = control group; PQ = Paraquat (alone)-treated group; PQ+PSE = PQ-induced group treated with PSE; PQ+PJ = PQ-induced group treated with PJ. Data are expressed as mean ± S.D. of 10 mice in each group. **: *P* < 0.01, ***: *P* < 0.001, and ****: *P* < 0.0001 compared with control group; #: *P* < 0.05, ##: *P* < 0.01 compared with PQ group

ATP level, the cell energy marker, significantly declined in the striatum after PQ injection when compared with the saline-injected group (*P* < 0.01; Fig. 3a). Oral administration of either PSE or PJ increased ATP levels significantly (*P* < 0.05) in relation to PQ (alone)-induced mice.

### Impact of PSE and PJ pretreatment on the striatal MDA and antioxidant enzyme activities in PQ-treated mice

PQ administration elevated significantly the striatal LPO degree represented by the increase of MDA level (*P* < 0.0001; Fig. 3b) when compared with the control group. Treatment of PQ-induced mice with PSE and PJ led to a remarkable decrease in MDA levels (*P* < 0.01 and *P* < 0.05, correspondingly; Fig. 3b) in relation to the PQ-treated group.

PQ injection induced a significant depletion in SOD, GPx, and CAT activities (*P* < 0.0001; Fig. 3c-e) when compared with the control group. Treatment of PQ-induced mice with PSE or PJ led to a substantial increase in the striatal activities of SOD, GPx, and CAT (*P* < 0.05) except for PJ effect on CAT activity that exhibited a non-remarkable difference when compared with the PQ (alone)-treated group (Fig. 3c-e).

### Effect of PSE and PJ on the striatal DNA fragmentation in PQ-injected animals

**Fig. 4:**
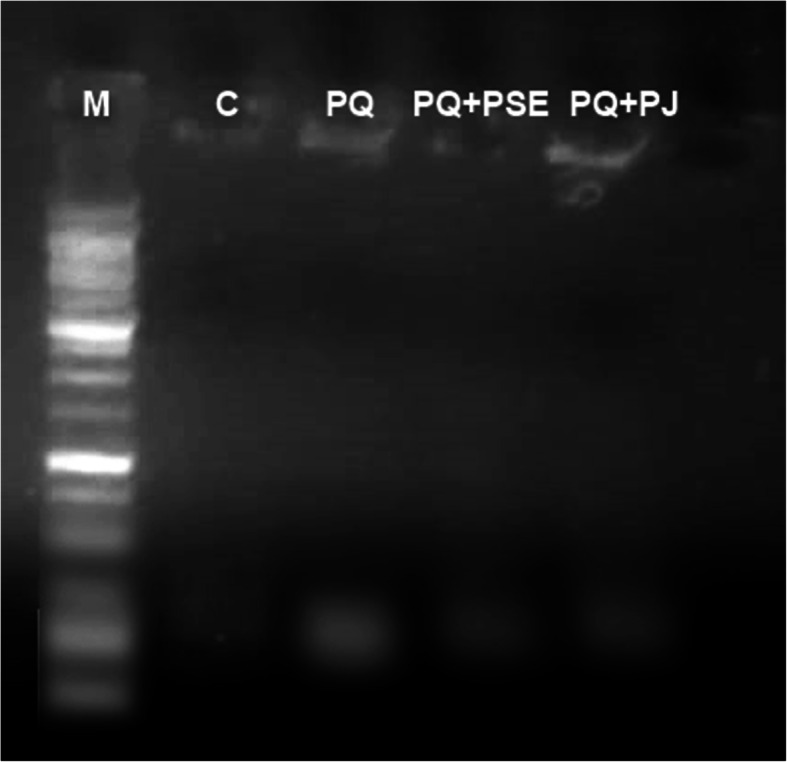
Representative cropped DNA electrophoresed patterns showing genomic DNA isolated from the striatum region of brain tissues of different animal groups. The full length gel is presented in supplementary figure 2. Lane (M) DNA marker. Lane (C) DNA of normal control group. Lane (PQ) smeared and laddered DNA in PQ (alone)-treated group. Lane (PQ+PSE) slight smear of PQ-induced group treated with PSE. Lane (PQ+PJ) slight smear of PQ-induced group treated with PJ. The assay was repeated at least three times

The current experiment showed that treatment with PQ induced apoptotic DNA fragmentation indicated by the appearance of fragmentized DNA in the form of both laddered and smeared DNA fragments when compared with the intact pattern of genomic DNA of the control group (Fig. 4). PQ-induced DNA damage was markedly reduced by the administration of both PSE and PJ as indicated by the existence of a slight smear of DNA with no apoptotic bands on the gel.

### Effect of PSE and PJ supplementation on the inflammatory cytokine levels in PQ-induced mice

**Fig. 5:**
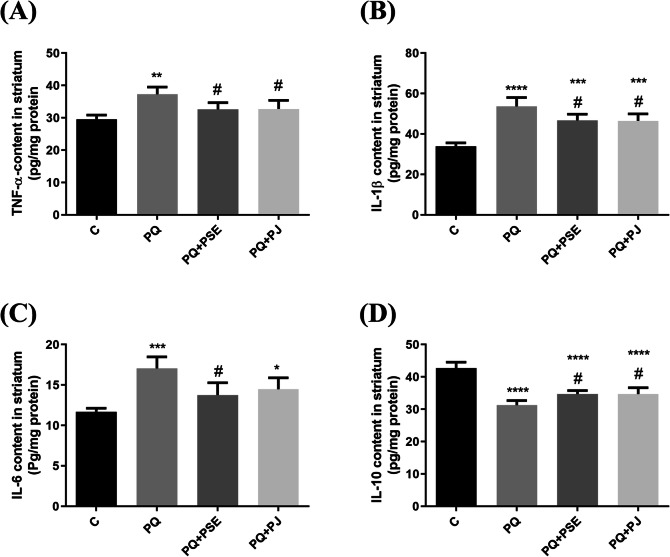
Effect of PSE and PJ supplementation on the striatal levels of pro-inflammatory and anti-inflammatory cytokines of PQ-treated mice. Striatal content of TNF-α, (**a**), IL-1β (**b**), IL-6 (**c**), and IL-10 (**d**). C = control group; PQ = Paraquat (alone)-induced group; PQ+PSE = PQ-induced group treated with PSE; PQ+PJ = PQ-induced group treated with PJ. Data are expressed as mean ± S.D. of 10 mice in each group.*: *P* < 0.05, **: *P* < 0.01, ***: *P* < 0.001, and ****: *P* <  0.0001 compared with control group; #: *P* < 0.05 compared with PQ group

Figure 5 shows the effect of PSE and PJ administration on TNF-α, IL-1β, IL-6, and IL-10 levels in striatal tissue of PQ-treated mice. There was a significant elevation in tissue pro-inflammatory cytokines; TNF-α, IL-1β, IL-6 in PQ (alone)-injected mice as compared with the normal control animals (*P* < 0.01, *P* < 0.0001, and *P* < 0.001, respectively; Fig. 5a-c). The level of the anti-inflammatory cytokine IL-10 reduced substantially in the PQ (alone)-treated group when compared with the normal group (*P* < 0.0001; Fig. 5d). Treatment of PQ-injected mice with PSE led to a marked decrease in the striatal levels of TNF-α, IL-1β, and IL-6 (*P* < 0.05, Fig. 5a-c); whereas IL-10 level increased significantly when compared with PQ (alone)-treated mice (*P* < 0.05; Fig. 5d). PJ supplementation significantly declined TNF-α, IL-1β levels (*P* < 0.05; Fig. 5a, b) with non-marked reduction of IL-6 level (Fig. 5c) in relation to PQ (alone)-treated mice. In addition, the level of IL-10 elevated significantly in the PQ + PJ group when compared with PQ (alone)-injected mice (*P* < 0.05; Fig. 5d).

### Effect of PSE and PJ administration on the striatal relative expression of NF-кB mRNA in PQ-treated animals

**Fig. 6:**
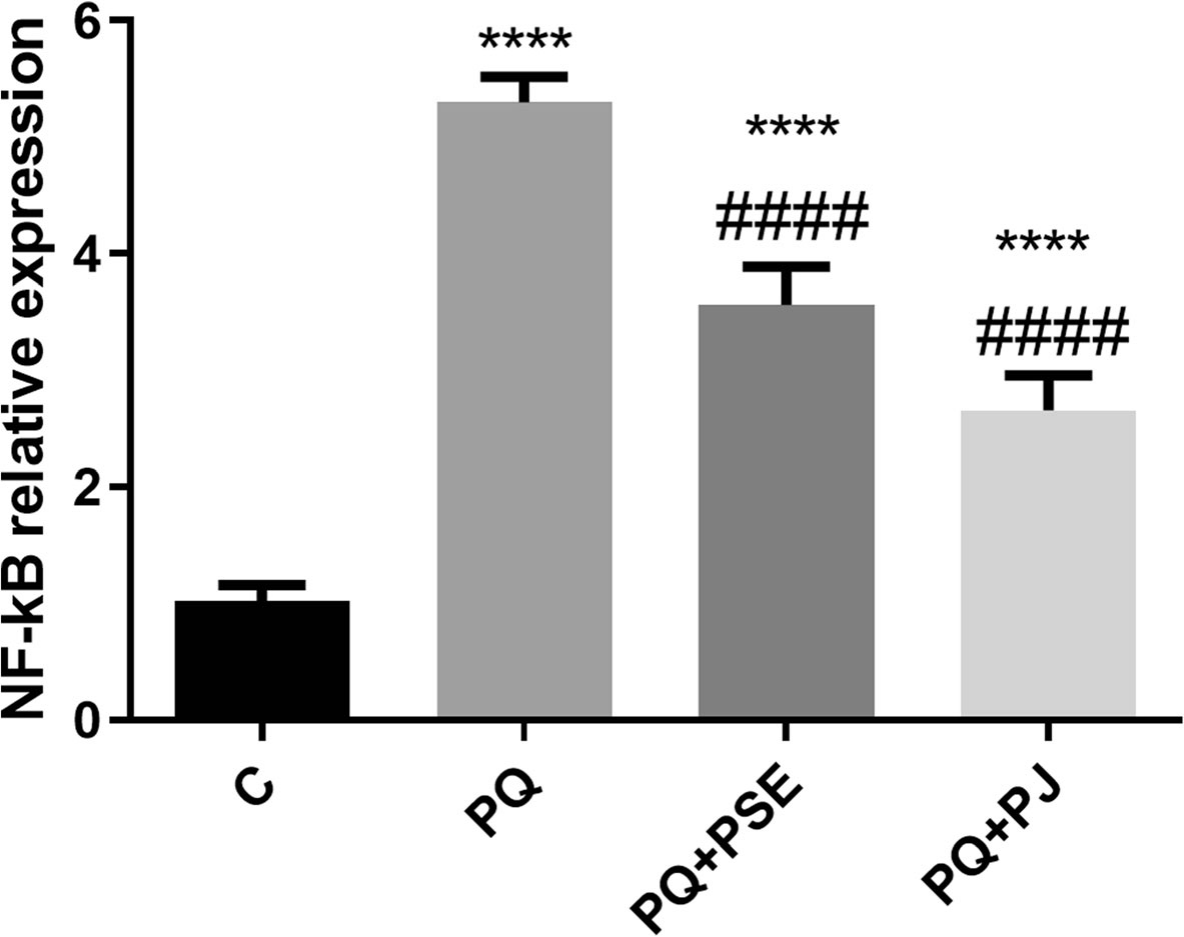
Effect of PSE and PJ supplementation on the striatal NF-кB gene expression in PQ-induced mice. C = control group; PQ = Paraquat (alone)-induced group; PQ+PSE = PQ-induced group treated with PSE; PQ+PJ = PQ-induced group treated with PJ. Data are expressed as mean ± S.D. of 10 mice in each group. ****: *P* < 0.0001 compared with control group; ####: *P* < 0.0001 compared with PQ group

Figure 6 showed variation in mRNA expression of NF-кB in the striatum of normal control, PD model of mice, and PD mouse model treated with either PSE or PJ. PQ injection significantly upregulated striatal NF-кB gene expression when compared with the control group (*P* < 0.0001). A significant downregulation of NF-кB gene expression was detected after oral administration of PSE or PJ, when compared with PQ (alone)-treated mice (*P* < 0.0001).

### Effect of PSE and PJ on trophic factors and microglial marker in the striatum of PQ-induced mice

**Fig. 7:**
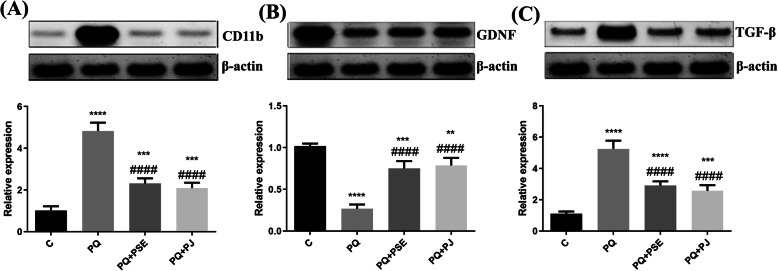
Representative cropped blots with relative expression levels of the striatal CD11b, TGF-β, and GDNF in different animal groups. The full length blots are presented in supplementary figure 3. Relative expression of CD11b (**a**), GDNF (**b**), and TGF-β (**c**) in striatum. C = control group; PQ = Paraquat (alone)-induced group; PQ+PSE = PQ-induced group treated with PSE; PQ+PJ = PQ-induced group treated with PJ. Data are expressed as mean ± S.D. of 10 mice in each group. **: *P* < 0.01, ***: *P* < 0.001, and ****: *P* < 0.0001 compared with control group; ####: *P* < 0.0001 compared with PQ group

Figure 7 reveals the modulation impact of PSE and PJ on certain striatal inflammatory markers. CD11b, TGF-β, and GDNF were measured. PQ injection significantly increased the levels of CD11b and TGF-β (P < 0.0001; Fig. 6a and b) when compared with the control group, while administration of either PSE or PJ into PQ-injected mice resulted in a substantial decline in CD11b and TGF-β levels (*P* < 0.0001; Fig. 7a and b) in relation to the PQ (alone)-treated group. Injection with PQ markedly reduced GDNF level when compared with the control mice (*P* < 0.0001; Fig. 7c), whereas PSE and PJ administration led to a significant elevation of GDNF level in relation to the PQ (alone)-treated group (*P* < 0.0001; Fig. 7c).

## Discussion

Different reports illustrated the relation between dietary supply and improved health problems. A close relation between vegetables and fruits’ intake with the reduced risk of various health disorders such as; diabetes, strokes, cancers, and neurodegenerative diseases, was elucidated [43]. Pomegranate (*Punica granatum* L.) contains variable phytochemicals that were referenced to exert antioxidant and anti-inflammatory impacts [22]. Although pomegranates have been shown to reveal positive impacts in numerous diseases [24], the protective activity of *Punica granatum* L. against the PQ-induced PD model has not been explored yet.

In the present article, TH level substantially reduced in the SN following PQ treatment while this decline was markedly prevented by PSE or PJ pretreatment. The reduction in TH protein and DA levels by different mechanisms might induce the propagation of the movement disorders during the process of dopaminergic cell death [5]. Subsequently, TH is believed to be a key protein involved in neurodegeneration in PD. [5] It has been previously shown that PQ injection (7 mg/kg) once a week for 4 weeks led to destroyed dopaminergic neurons in the SN [12]. In the current study, mice were treated with a higher dose (10 mg/kg) twice a week for 3 weeks, so it is likely that PQ impact might be associated with dopaminergic neuronal destruction. Subsequently, PSE and PJ pretreatment protected against nigral dopaminergic neuronal loss as revealed by the increased level of TH.

In the current study, the levels of DA and its metabolite, DOPAC, markedly reduced following PQ treatment. Treatment with PQ was reported to reveal variable impacts on DA and DOPAC levels in different brain regions [44]. However, our results were consistent with Kuter et al. [45] who recorded that DA level and DA metabolism were reduced in the striatum following PQ treatment. In the current study, administration of PSE and PJ revealed a protective potential against DA and DOPAC deficiency in the striatum of PQ-treated mice. The previous outcome may be attributed to the polyphenolic and other phytochemical constituents that were recorded in the GC-MS analysis of PSE and PJ. Our results were in agreement with Ortega-Arellano et al. [46] who recorded a protective effect of polyphenols against PQ in Drosophila.

ATP is an important indicator of the energy exchange and the functional integrity for all active cells and its measurement can be used as a clue for detecting dopaminergic neuronal loss [16]. It has been revealed in the current article that ATP level reduced in mice’ striatum after PQ injection which may be an indication for dopaminergic cell loss. Impairment in ATP level along with mitochondrial dysfunction had been confirmed in different animal models, in human individuals, and in-vitro studies of neurodegenerative diseases including PD. [43] PSE and PJ raised ATP level in the striatum. It has been reported that oxidative destruction of DNA, proteins, and membranes as well as the inability of the mitochondria to generate ATP are attributed to ROS [47]. Subsequently, it can be deduced that rescuing neuronal cells of the striatum and preserving the integrity as well as the normal metabolic functions of the striatal cells might be achieved via ROS squelching capabilities of PSE and PJ contents [43, 48].

Excessive accumulation of oxidatively damaged molecules or oxygen free radicals was prominent in various disorders, including neurodegenerative diseases such as PD. [43] In the present study, an increase of MDA in the striatum of PQ (alone)-induced mice was observed when compared with the normal control mice. MDA serves as an LPO indicator and is a crucial biomarker for oxidative stress [49]. An increased MDA level following PQ injection is consistent with the results of Ortiz et al. [49] who revealed a significant elevation of MDA level in the brain following PQ treatment suggesting that PQ injection induces peroxidative reactions. ROS liberation was confirmed nearly in all cells with superoxide anion radicals as the vital source during oxidative phosphorylation. Hydrogen peroxide is another ROS that is formed due to the conversion of superoxide by the action of superoxide dismutases (SODs). Although hydrogen peroxide was proven to be detoxified by CAT as well as by GPx enzymes, it can be converted by the Fenton reaction to hydroxyl radicals that can destroy membrane lipids with lipid peroxidation outcome [43]. PSE and PJ phytochemicals, the total phenolics, and the total flavonoids might have antioxidant properties to counteract the MDA increment and to protect the striatum from the oxidative stress. This outcome is in agreement with Guo et al. [21] who attributed the MDA decline and the rescued oxidative stress in PD models to the polyphenolic constituents of pomegranate fruit. An important consequence of PD is the induced oxidative stress due to the accumulation of ROS as well as the disturbing activity of the antioxidant enzymes such as SOD, CAT, and GPx. The current study revealed that PQ treatment reduced the antioxidant enzyme activities which might be ascribed with the excess production of ROS with an inability to be squelched by the disturbed antioxidant enzymes following PQ injection. PSE and PJ pretreatment protected the antioxidant defense system and enhanced the activity of the antioxidative enzymes in the striatum of PQ-treated mice. The neuroprotective effects of different flavonoids in neurodegenerative disorders were confirmed in various in-vivo and in-vitro studies due to their abilities to get rid of oxygen free radicals [43].

Apoptosis or programmed cell death was potentiated following PQ administration into *Drosophila melanogaster* via the oxidative stress-induced pathway [46]. The molecular cascades for apoptosis were inferred to ROS production and chromatin condensation/DNA fragmentation [49]. Moreover, Apoptotic induction was demonstrated following the MDA elevated release [43]. In the present work, DNA fragmentation was observed in the striatum following PQ treatment. Our result was in line with Chaudhuri et al. [50] who showed impaired DNA of the dopaminergic neurons by activated oxidative stress and mitochondrial destruction after injection with PQ in *Drosophila melanogaster*. In the present experiment, PSE and PJ constituents preserved the neuronal structure in the striatum by prohibiting chromatin condensation. The abovementioned effect might be attributed to the counteracting antioxidant phytochemical components along with the total phenolic and flavonoids that were detected in both PSE and PJ.

Different chronic diseases including neurodegenerative pathologies are correlated with the activation of chronic inflammation [51] with persistently induced components of the innate and adaptive immune system. Microglial cells, macrophages of the central nervous system, were proven to be recruited at the specific lesion site of various neurodegenerative disorders with ROS and pro-inflammatory cytokine secretion that destroy the cells [43]. In the present study, the levels of the striatal pro-inflammatory cytokines; TNF-α, IL-1β, IL-6, and the striatal microglial marker; CD11b elevated significantly in the PQ (alone)-injected animals. Meanwhile, an anti-inflammatory mediator; IL-10 decreased in the striatum of PQ (alone)-treated mice. Our results were in agreement with Stojkovska et al. [52] who revealed increased levels of the pro-inflammatory cytokines following PQ treatment. Neuroinflammation has been confirmed to play a vital role in the propagation of PD pathology. Microglial activation with increased secretion of the pro-inflammatory cytokines has been reported in different PD-induced animal models [53]. Cytokines, chemokines, and ROS secretions via microglial mediation were also confirmed to be the main contributors to a sustained immune response during neuroinflammation [16]. PD was confirmed to activate the inflammatory response with microglial induction which predisposes for neurodegenerative consequences of the disease [47]. As revealed in the present study, PSE and PJ pretreatment protected the striatal cells from the released pro-inflammatory cytokines and induced the secretion of the anti-inflammatory cytokine; IL-10. The previous outcome may be attributed to the polyphenolic compounds that were detected in PJ and PSE. Similarly, polyphenolic compounds of grape seed extract exhibited an anti-inflammatory impact in the brain of Alzheimer’s disease transgenic mouse model [54]. The anti-inflammatory effects of flavones have been also demonstrated in animal models of multiple dietary trials [55].

Activation of the transcription factor; NF-кB elicits an inflammatory response and implies neuronal integrity and signaling [56]. In the present work, NF-кB upregulation was shown in the striatum of PQ (alone)-treated mice. The previous outcome was in line with Chen et al. [57] who revealed a similar finding with PQ supplementation into murine embryonic fibroblasts isolated from mice. It has been reported that the pro-inflammatory NF-кB transcription factor is very sensitive towards oxidative stress [58] and can be activated by ROS either directly or indirectly through induction of pro-inflammatory cytokine release [57]. For further support, this vital transcription factor was proven to be activated in response to the inflammatory cytokines and ROS after traumatic brain injury [56].

Besides, NF-кB activation has an essential pro-apoptotic role in PD and was correlated with the dopaminergic cell destruction in the 6-hydroxydopamine (6-OHDA) induced PD model of rats [59]. Consequently, it can be assumed that prohibiting NF-кB activation either through inhibition of oxidative stress mechanism or pro-inflammatory mediators’ release conveys a protective effect against PD pathology [59]. PSE and PJ showed a safeguarding effect since NF-кB activation was suppressed by their phytochemical constituents. The previous effect can be attributed to the protective effect of phytochemicals especially the polyphenolic compounds against ROS formation and pro-inflammatory cytokine release. It has been reported that the survival rate of dopaminergic neurons was enhanced in dissociated primary cell culture treated with red grape seed and skin extract through reducing NF-кB pathway as well as diminishing oxidative stress and apoptosis [55]. Moreover, it was proven that isovitexin treatment downregulated NF-кB in lipopolysaccharide-induced RAW264.7 macrophages [60].

The current results demonstrated the levels of trophic factors; TGF-β and GDNF in PQ-induced mice and their levels after treatment with either PSE or PJ. The level of TGF-β significantly increased while GDNF substantially declined after PQ treatment. It has been reported that TGF-β is expressed in the dopaminergic system pathway since the embryonic stage of development and its mRNA is upregulated as a result of the exposure to different lesions [61]. Contradictory results upon TGF-β signaling activation were recorded in various disease models which in turn indicate the complexity of this factor activation as a double-edged sword in brain pathology. It has been stated that TGF-β which was well known as an anti-inflammatory cytokine following a stroke, worked as a pro-inflammatory factor in models of autoimmune encephalitis [62, 63]. Moreover, astrocytic dysfunction and exacerbated inflammation were correlated to TGF-β signaling induction in epileptogenesis [64, 65]. Despite many trophic factors were used effectively in in-vivo and in-vitro models for PD, TGF-β effects in neurons are contextual and can affect the survival of neuronal cells either positively or negatively depending on different factors such as the released cytokines [61]. Noteworthy, the upregulation of NF-кB genes was induced via TGF-β signaling by albumin [64]. It has been reported that TGF-β activated the arrest of growth and induced apoptosis in primary rat oligodendrocyte progenitor culture [65]. Subsequently, the DNA fragmentation of striatal cells following PQ treatment might be augmented with an increased level of TGF-β. For further support, TGF-β was proven to act synergistically with TNF-α to induce cell death and apoptosis in Schwann cells while each of these factors cannot reveal that effect alone [66]. Therefore, it can be inferred that TGF-β might exert a pro-inflammatory effect and with the increased level of TNF-α, it revealed a pro-apoptotic impact on striatal neurons following PQ treatment. Consequently, PSE and PJ succeeded to rescue striatal cells from the induced pro-apoptotic impact of PQ via reducing TGF-β.

It was recorded that GDNF is an important factor for dopaminergic neuronal survival [18] and that the elevated level of GDNF could induce the function of dopaminergic neurons and might exhibit a neuroprotective role in PD animal models [53]. The results in the current experiment showed that both PSE and PJ increased the level of GDNF in the striatum of PQ-injected animals. The abovementioned effect may be due to the presence of certain polyphenolic constituents in PSE and PJ which increased the level of this neuroprotective factor [43].

Polyphenols include various groups that were classified based on the number of phenol rings and the chemically attached groups to them. The most crucial group of polyphenols is named as flavonoids that contain compounds such as vitexin, isovitexin, and flavones. The abovementioned components were recorded in the present work in PSE and PJ (Tables 1 & 2). Other polyphenols were also detected by the GC-MS analysis such as propyl gallate, nobiletin, and ellagic acid in PSE, ellagic acid in PJ. Moreover, triterpenoids, isoprenoids, unsaturated fatty acids, and primary fatty alcohols were exhibited in GC-MS results of either PSE or PJ or both. Collectively, the polyphenols and other phytochemical constituents that were detected in PSE and PJ have been reported to intimately exhibit neuroprotective impacts via their antioxidative, anti-inflammatory, and anti-apoptotic consequences [22, 46, 48, 55, 60].

## Conclusions

In conclusion, PQ injection supressed the TH level in the SN which confirms the hallmark feature of PD that was prevented upon pretreatment with either PSE or PJ. Furthermore, oxidative damage effect following PQ treatment might promote inflammatory reaction which induced DNA fragmentation with the decreased DA and DOPAC secretions along with the reduced ATP level in the striatum. Treatment with either PSE or PJ showed a protective impact against PQ-induced toxicity in the striatum through protection from the oxidative stress, inhibition of inflammation, rescuing the cells from DNA fragmentation, and restoration of ATP level. Thus PSE and PJ supplementation may be useful therapeutic agents that protect from PD.

## Supplementary Information

**Additional file 1.**

**Additional file 2.**

**Additional file 3.**

## Data Availability

The authors declare that the data supporting the findings of this study are included within the article [and/or] its supplementary materials.

## Abbreviations

ATP: Adenosine triphosphate
CAT: Catalase
CD11b: Cluster of differentiation 11b
CAIM: Herbarium of Flora Researches Centre, Agriculture museum campus
DA: Dopamine
DOPAC: 3,4-dihydroxyphenylacetic
ELISA: Enzyme-linked immunosorbent assay
GAPDH: Glyceraldehyde 3-phosphate dehydrogenase
GC-MS: Gas chromatography–mass spectrometry
GDNF: Glial cell-derived neurotrophic factor
GPx: Glutathione peroxidase
HPLC: High-performance liquid chromatography
IL-1β: Interleukin-1β
IL-6: Interleukin-6
IL-10: Interleukin-10
LBs: Lewy bodies
MDA: Malondialdehyde
NF-кB: Nuclear factor kappa B
NIH: National Health Institute
NODCAR: National Organization for Drug Control and Research
6-OHDA: 6-hydroxydopamine
PBS: Phosphate buffer saline
PD: Parkinson’s disease
PJ: Pomegranate juice
PSE: Pomegranate seed extract
PQ: Paraquat
PVDF: Polyvinylidene difluoride membranes
qPCR: Real-time polymerase chain reaction assay
ROS: Reactive oxygen species
SNpc: Substantia nigra pars compacta
SOD: Superoxide dismutase
TBARS: Thiobarbituric acid reacting substances
TGF-β: Transforming growth factor-β
TH: Tyrosine hydroxylase
TNF-α: Tumor necrosis factor
TFC: Total flavonoid content
TPC: Total phenolic content
