# Supplementary figures and images for: Neuroprotective effects of pomegranate (Punica granatum L.) juice and seed extract in paraquat-induced mouse model of Parkinson’s disease
Source: BMC Complement Med Ther. 2021 Apr 26;21:130. doi: 10.1186/s12906-021-03298-y (PMC8074500; doi:10.1186/s12906-021-03298-y)

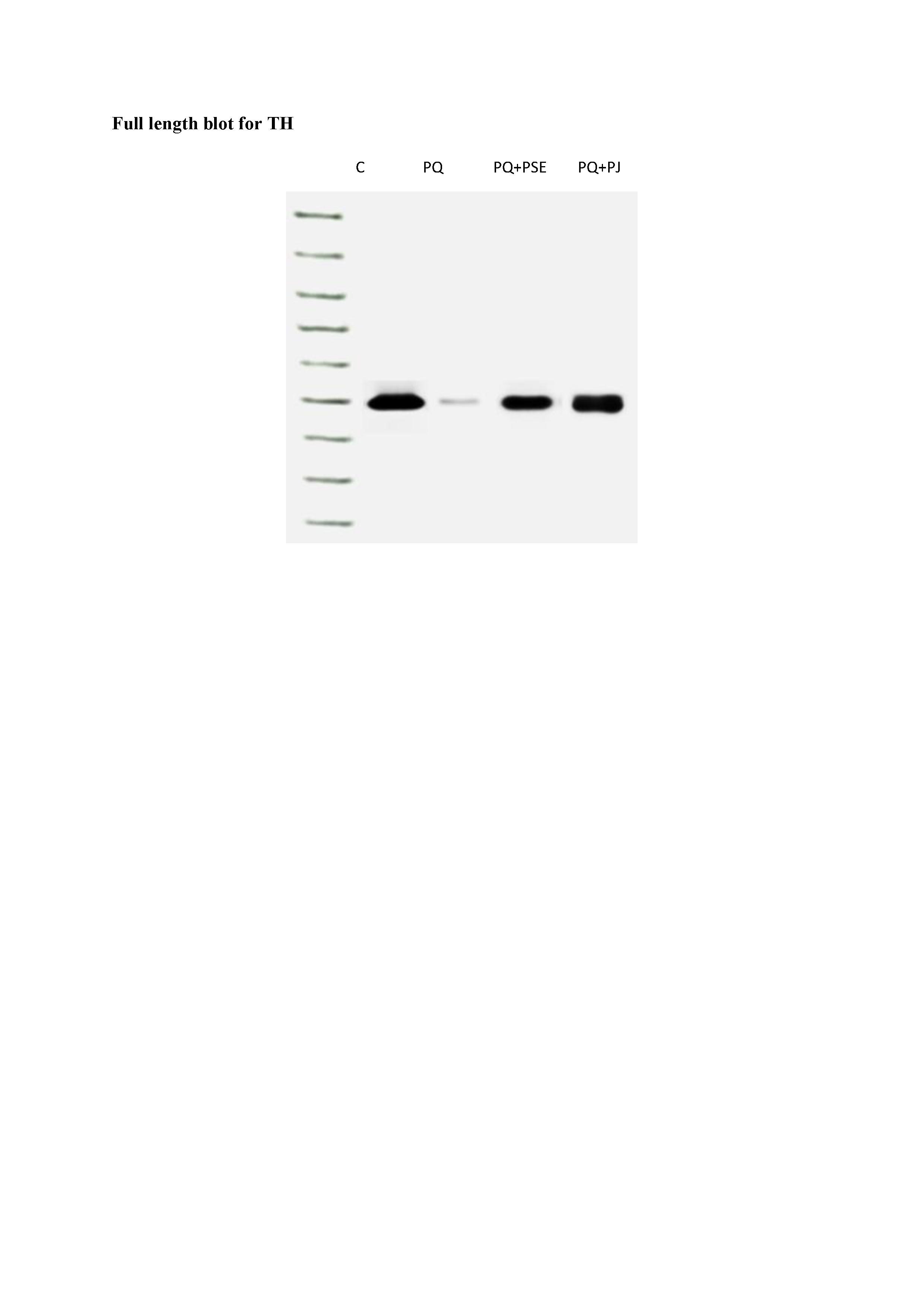

Supplement: Supplementary file 1 — Additional file 1. [file 12906_2021_3298_MOESM1_ESM.tif]

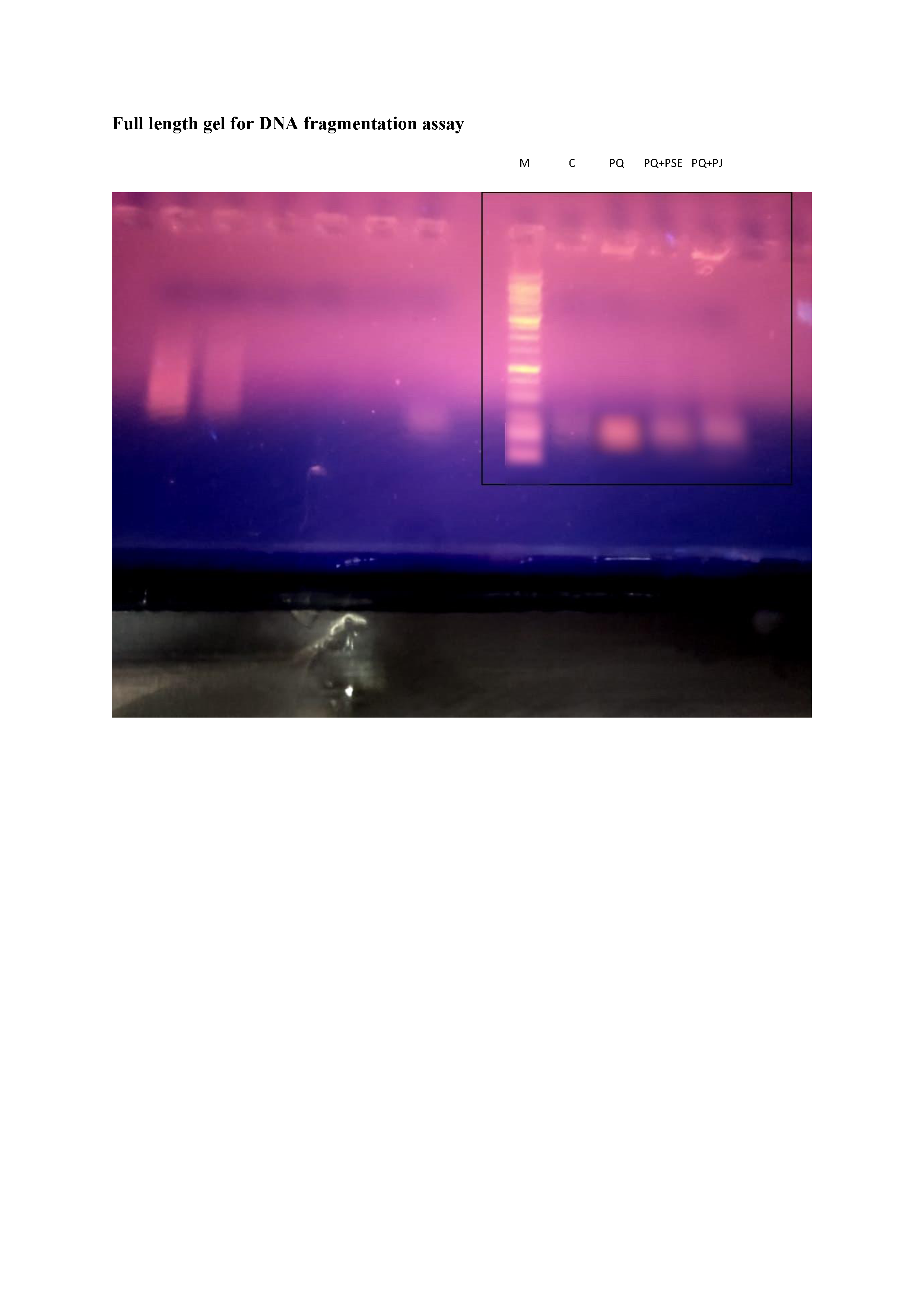

Supplement: Supplementary file 2 — Additional file 2. [file 12906_2021_3298_MOESM2_ESM.tif]

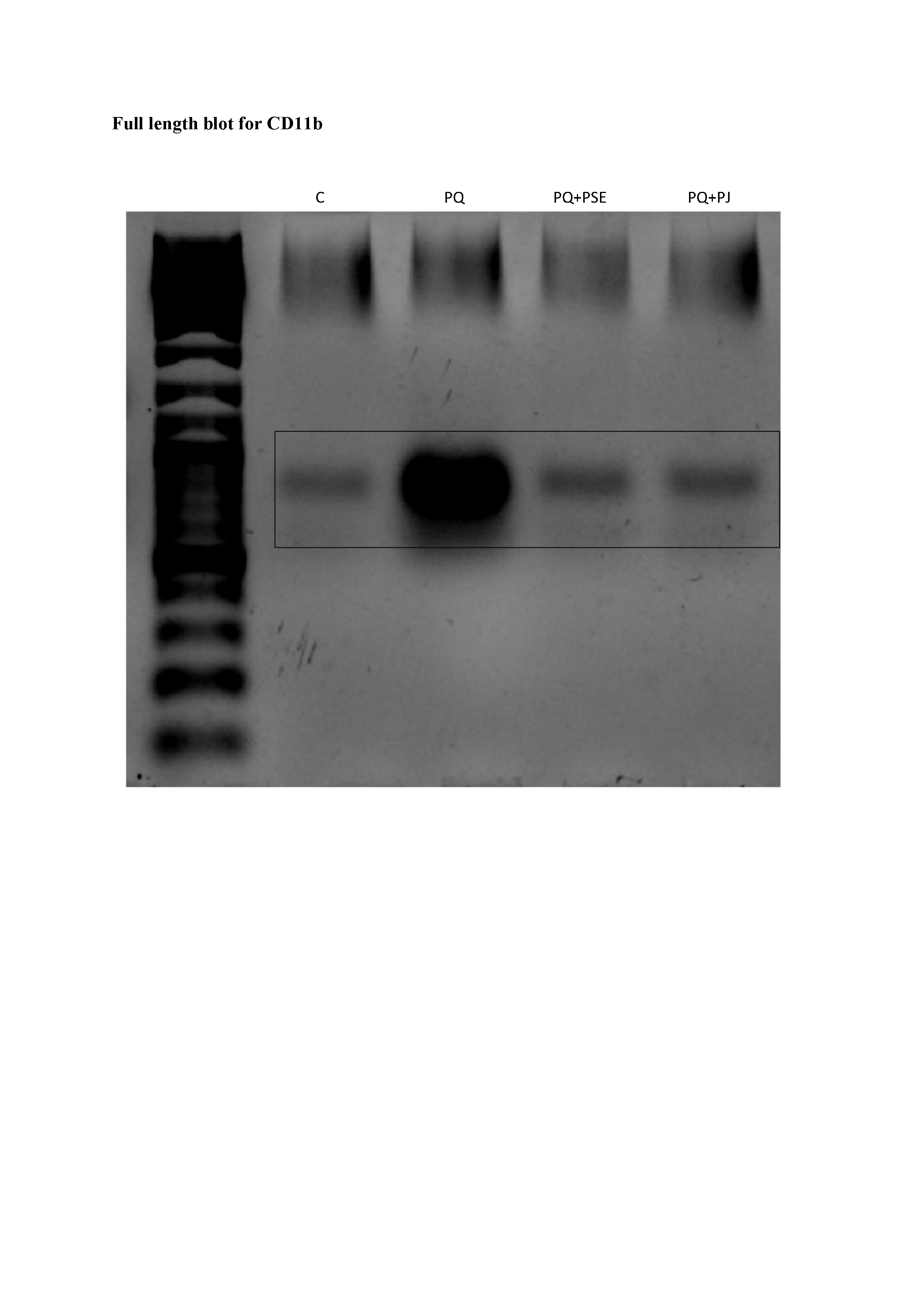

Supplement: Supplementary file 3 — Additional file 3. [file 12906_2021_3298_MOESM3_ESM.tif]
